# Nitric Oxide-Mediated Modulation of Central Network Dynamics during Olfactory Perception

**DOI:** 10.1371/journal.pone.0136846

**Published:** 2015-09-11

**Authors:** Satoshi Watanabe, Fumihito Takanashi, Kohei Ishida, Suguru Kobayashi, Yoshiichiro Kitamura, Yuuta Hamasaki, Minoru Saito

**Affiliations:** 1 Department of Bioengineering and Robotics, Graduate School of Engineering, Tohoku University, Sendai, Japan; 2 Graduate School of Pharmaceutical Sciences, University of Tokyo, Tokyo, Japan; 3 Department of Correlative Study in Physics and Chemistry, Graduate School of Integrated Basic Sciences, Nihon University, Tokyo, Japan; 4 Kagawa School of Pharmaceutical Sciences, Tokushima Bunri University, Sanuki, Japan; 5 College of Science and Engineering, Kanto Gakuin University, Yokohama, Japan; Monell Chemical Senses Center, UNITED STATES

## Abstract

Nitric oxide (NO) modulates the dynamics of central olfactory networks and has been implicated in olfactory processing including learning. Land mollusks have a specialized olfactory lobe in the brain called the procerebral (PC) lobe. The PC lobe produces ongoing local field potential (LFP) oscillation, which is modulated by olfactory stimulation. We hypothesized that NO should be released in the PC lobe in response to olfactory stimulation, and to prove this, we applied an NO electrode to the PC lobe of the land slug *Limax* in an isolated tentacle-brain preparation. Olfactory stimulation applied to the olfactory epithelium transiently increased the NO concentration in the PC lobe, and this was blocked by the NO synthase inhibitor L-NAME at 3.7 mM. L-NAME at this concentration did not block the ongoing LFP oscillation, but did block the frequency increase during olfactory stimulation. Olfactory stimulation also enhanced spatial synchronicity of activity, and this response was also blocked by L-NAME. Single electrical stimulation of the superior tentacle nerve (STN) mimicked the effects of olfactory stimulation on LFP frequency and synchronicity, and both of these effects were blocked by L-NAME. L-NAME did not block synaptic transmission from the STN to the nonbursting (NB)-type PC lobe neurons, which presumably produce NO in an activity-dependent manner. Previous behavioral experiments have revealed impairment of olfactory discrimination after L-NAME injection. The recording conditions in the present work likely reproduce the in vivo brain state in those behavioral experiments. We speculate that the dynamical effects of NO released during olfactory perception underlie precise odor representation and memory formation in the brain, presumably through regulation of NB neuron activity.

## Introduction

Olfactory signals are transmitted from olfactory sensory neurons in the olfactory epithelium to olfactory centers via fast excitatory synapses, but besides these specific transmissions, olfactory processing is modulated both centrally and peripherally by slow diffusive neuromodulatory systems. Widespread neuromodulatory systems including cholinergic and monoaminergic neurons may mediate context-dependent olfactory processing in the network, such as modification of sensitivity and discriminability by arousal state [1], satiety [2] and olfactory learning [3], and may be related to pathological conditions [4,5]. Gaseous neurotransmitters are another category of neuromodulators, and in olfactory systems, this category includes nitric oxide (NO) and carbon monoxide (CO) [6]. The highly mobile character of gaseous transmitters causes widespread and relatively rapid modulation of a wide range of networks.

NO is synthesized from L-arginine by NO synthase (NOS), and the major NOS subtypes found in neural systems are activated by calcium/calmodulin and modulate target molecules (such as ion channels) in an activity-dependent manner, either directly or through activation of guanylyl cyclase [7]. NO modulates neural activity and synaptic transmission in olfactory systems [8–11]. The functions of NO has been well documented in relation to learning, both in vertebrates [12–14] and invertebrates [15–22].

In the land slug *Limax*, accumulating evidence suggests that NO functions specifically in the procerebral (PC) lobe, which is the olfactory center essential for olfactory learning; it shows unique network activity including ongoing local field potential (LFP) oscillations [23,24]. The *Limax* homologs of NOS have been cloned [25,26]. The expression levels of NOS genes and soluble guanylyl cyclase and the activity of NADPH diaphorase are all high in the PC lobe [6,26–28] Exogenous application of an NO donor or uncaging of caged NO strongly increases the frequency of the ongoing LFP oscillations and depolarizes the bursting (B) neurons [6,29,30]. Behavioral studies using an NO synthase blocker showed impairment of appetitive olfactory learning [31] and olfactory discrimination after aversive learning [32,33].

Although the functional significance of ongoing LFP oscillation and its modulation has not been fully elucidated, it has several prominent characteristics. First, the oscillation frequency is strongly increased by olfactory stimuli [34]. Second, the LFP oscillation has a phase gradient along the apex-base axis. During each cycle of oscillation, activity propagates from the apex to the base, and this forms traveling waves. The speed of the traveling wave increases during olfactory stimulation, resulting in nearly synchronous activity [35]. Third, stimulus-evoked modulation of the LFP oscillation is learning-dependent [36,37]. Although the odor-evoked modulatory effects are similar to those of exogenous NO and may suggest dynamical, as opposed to biochemical, regulation of physiological functions by NO, there has been neither direct evidence for actual NO release in response to olfactory input, nor has it been shown that NO is the cause of odor-induced modulations of neural activity in the PC lobe.

In the present work, we use an NO electrode to detect NO release in the PC lobe. NO electrodes have been used in vertebrate brains in vivo and in slices [38], as well as in invertebrate neurons [39–41]. We then apply an NO synthase inhibitor to prove stimulus-evoked release of NO in olfactory responses. Our results show modulation of multiple parameters of the dynamics by a single gaseous transmitter, which might be critical for adaptive olfactory processing.

## Materials and Methods

### Preparation and odor stimulation

The slugs *Limax valentianus* were from the laboratory colonies kept at the University of Tokyo, Nihon University, Tokushima Bunri University and Fukuoka Women's University. We dissected out the CNS together with a superior tentacle (tentacle-brain preparation) from the animals of 11–14 weeks post-hatching and weighing 0.2–0.4 g [42]. The preparation was fixed in a recording chamber which had separate compartments for the brain and the tentacle. The brain compartment was filled with saline (70 mM NaCl, 2 mM KCl, 4.9 mM CaCl_2_, 4.7 mM MgCl_2_, 5 mM glucose, 5 mM HEPES, pH 7.2). The tentacle compartment was initially filled with saline. The preparation was left to recover for 1 hr before recording. All recordings were made at room temperature (20−24°C).

Before recording, saline was removed from the tentacle compartment and a constant air puff (about 8 ml/min) was directed at the olfactory epithelium at the tentacle tip. Odor-containing air was puffed for 15 s by switching the air flow using electric valves. 1-hexanol (hexanol), 2-ethyl-3-methoxypyrazine (EMOP) and garlic were used as odorants. Hexanol and EMOP were diluted with liquid paraffin. Garlic was grated and stored at −20°C until use. Odorant was placed in the air path for odor stimulation. After the first recording, the tentacle compartment was filled with saline again, and the bath solution in the brain compartment was replaced by saline containing either L-NAME or its inactive enantiomer D-NAME (Wako Pure Chemical, 3.7 mM). Ater 90 min, a second recording was made.

### NO electrode

In order to monitor NO release by olfactory stimulation, we used an NO recording system (NO-501, Inter Medical) with an NO electrode (NOE-10W, Bio Research Center) and a reference electrode (NOR-20, Bio Research Center). Before recording, the NO electrode was calibrated as follows. First, the electrode was immersed in N_2_-saturated saline solution, which had been bubbled with N_2_ for about 5 min. This gives the basal output of the electrode (*A*_1_). Then the saline solution was saturated with NO (100 ppm, balanced with N_2_) and the output was recorded (*A*_2_). Finally, the saline solution was saturated with N_2_ and the basal output was recorded again (*A*_3_). The baseline-subtracted output *A* was calculated as *A* = *A*_2_ − (*A*_1_+*A*_3_)/2. The NO concentration in the solution was given as *C*_NO_ = (*P*_a_−*P*_H2O_) *G*_NO_ *α* /*RT*, where *P*_a_ is the atmospheric pressure, *P*_H2O_ is the vapor pressure of water (2.34 kPa at 20°C), *G*_NO_ is the concentration of the bubbled NO during the calibration (100 ppm in the present work), *α* is the solubility of NO (0.047 at 20°C), *R* is the gas constant (8.314 J/K mol), and *T* is the absolute temperature. Dividing *C*_NO_ by *A* gives the coefficient of conversion. Our calibration gave 3.64±0.22 nM/pA (mean±SEM, N = 14), which was close to previously reported data using the same system: 4.0 nM/pA [40] and 1.5 nM/pA [43]. The typical response to 100 ppm NO was about 10 pA. If an atypical current was recorded, the electrode was discarded.

The NO electrode was placed in the internal mass of the PC lobe, and the NO response to odor stimulation was recorded. To allow access to the electrode, the sheath over the internal mass was removed. The data were digitized at 100 Hz and 16 bit resolution using a data acquisition board (PCI-6221, National Instruments) and LabView (National Instruments). Igor Pro (Wavemetrics) was used for analysis of the data. To cancel the deviation of the baseline, which mainly arises from a slow recovery process after an artifact during manipulation of the preparation, the baseline was fitted by an exponential curve and subtracted from the data. The data were temporally binned by 2 s and converted to NO concentration by multiplying with the coefficient of conversion.

### LFP and perforated patch recording, imaging, and statistical tests

We recorded the LFP from the posterior surface of the PC lobe using glass electrodes filled with saline. The electrode was placed near the apical end of the PC lobe. When a dual recording was made, the second electrode was placed near the center of the PC lobe. The signal was amplified, band-pass filtered at 0.5−30 Hz (0.5−300 Hz when electrical stimulation was applied) by an amplifier (MEG-2100, Nihon Kohden) and recorded using a data acquisition board (PCI-6221, National Instruments) and Igor Pro. The data were digitized at 1 kHz and 16 bit resolution. The instantaneous LFP frequency was calculated as the inverse of the interval between the two consecutive LFP peaks. To calculate the normalized LFP frequency change by olfactory stimulation, the average frequency change of the three LFP cycles showing the maximal response was divided by the resting frequency. For calculation of the normalized frequency change by STN stimulation, the average frequency change of the four cycles following the stimulation was divided by the resting frequency. For calculation of the normalized decrease in the phase lag by STN stimulation, the lag of the first LFP event after the stimulation was divided by the resting lag, and this was subtracted from 1.

Perforated patch recording was made in the nonbursting (NB) neurons as previously described [44,45]. The electrode contained 35 mM K gluconate, 35 mM KCl, 5 mM MgCl_2_, 250 μg/ml nystatin, 5 mM HEPES, pH 7.2. Axopatch 200B (Axon Instruments) and pCLAMP software were used. The data were analyzed using MATLAB (Mathworks).

Optical recording of the membrane potential was made in an isolated tentacle-brain preparation which was stained with 86 μM Di-4-ANEPPS (Sigma) for 50 min. Images were acquired using an upright microscope (E-FM1, Nikon) with a 16x water-immersion objective (NA = 0.8) and a sCMOS camera (Zyla, Andor) at 100 frames/s and 16-bit A/D conversion. Regions of interest (ROIs) of 32 μm square were set at the apical and basal sites of the PC lobe. The average pixel values of the ROIs were smoothed with a Savitzky-Golay filter and divided by the time-averaged values to derive the fractional changes (ΔF/F) using a custom program of MATLAB. The fluorescence traces were inverted in the figures. For calculation of the normalized decrease in the phase lag by olfactory stimulation, the average lag at two consecutive events showing the maximal decrease was divided by the resting lag, and this was subtracted from 1.

Electrical stimulation of the STN was made from the cut end using a suction electrode connected to an isolator (AMPI). Voltage (3–5 V) pulses of 1 ms duration were applied. Because the effect of STN stimulation depends on the timing relative to the LFP oscillation [46], stimulation was delivered in the middle of the interval between LFP peaks, or IPSP troughs (in the case of perforated patch recording).

For the comparison of the effects of L-NAME and D-NAME, a two-way mixed model ANOVA was used followed by paired t-tests with Bonferroni correction. For statistical tests of the related data, paired t-test was used. For independent data, unpaired t-test was used. For analysis of the correlation between two variables, Spearman's rank correlation was used. P values less than 0.05 were assumed to be significant. Error bars in the figures represent the SEM.

## Results

**Fig 1:**
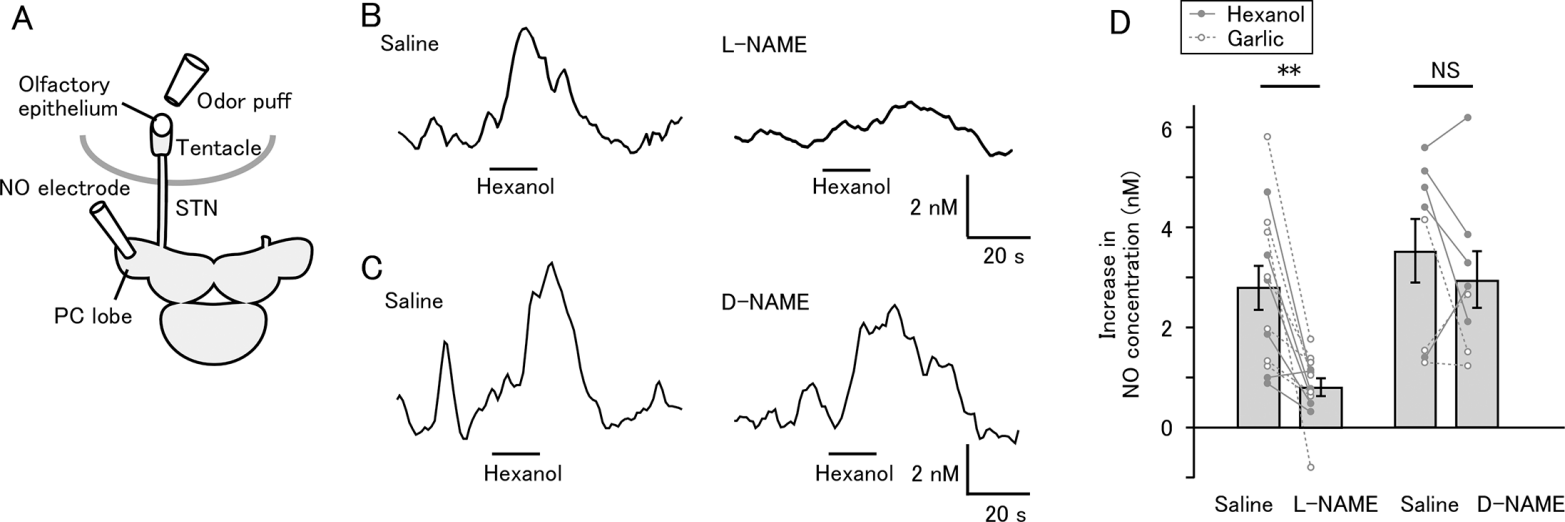
Odor stimulation triggers release of NO in the PC lobe. (A) Schematic of the isolated tentacle-brain preparation used for NO measurement. NO concentration was recorded using an NO electrode placed in the PC lobe. The tentacle was connected to the brain by the superior tentacle nerve (STN). Odorant (0.001% hexanol or garlic) was applied to the olfactory epithelium. (B) Odor-evoked increase in NO concentration in the PC lobe. After application of L-NAME, the response became smaller. (C) Effect of D-NAME on odor-evoked increase in NO concentration. The response after application of D-NAME was similar to the response in normal saline. (D) Summary of NO increase in response to olfactory stimulation before and after application of L-NAME or D-NAME. Average and individual data points are shown. L-NAME significantly reduced the NO increase (**P<0.01; N = 13), while D-NAME had no significant effect (NS, not significant; N = 8).

We monitored NO release in the PC lobe in an isolated tentacle-brain preparation by an NO electrode (Fig 1A). Air flow was directed at the olfactory epithelium while the brain was in control saline. When the air was switched to an odorant (0.001% hexanol or garlic), the NO concentration in the PC lobe increased by about 2 nM, and after termination of the stimulation, it returned to the pre-stimulus level. Hexanol and garlic produced similar elevations in the NO concentration (hexanol 3.29±0.54 nM, N = 11; garlic 2.85±0.51 nM, N = 10; unpaired t-test, P = 0.553), and the data with hexanol and garlic were combined. Next, we replaced the bath solution with saline containing either L-NAME or D-NAME and repeated the olfactory stimulation. Two-way mixed model ANOVA revealed a significant between-group difference between the L-NAME and D-NAME groups (P = 0.0127). Post-hoc tests revealed that the response to odor was significantly reduced by L-NAME (Fig 1B and Fig 1D, left; mean±SEM: saline 2.80±0.43 nM, L-NAME 0.81±0.17 nM, paired t-test with Bonferroni correction, P = 0.0011, N = 13 (6 with hexanol and 7 with garlic)), but not by D-NAME (Fig 1C and Fig 1D, right; saline 3.53±0.71 nM, D-NAME 2.96±0.57 nM, P = 0.706, N = 8 (5 with hexanol and 3 with garlic)). For the L-NAME group, Cohen’s d value was 1.68. These results suggest that the signal recorded by the NO electrode arises from NO released in response to olfactory stimulation.

**Fig 2:**
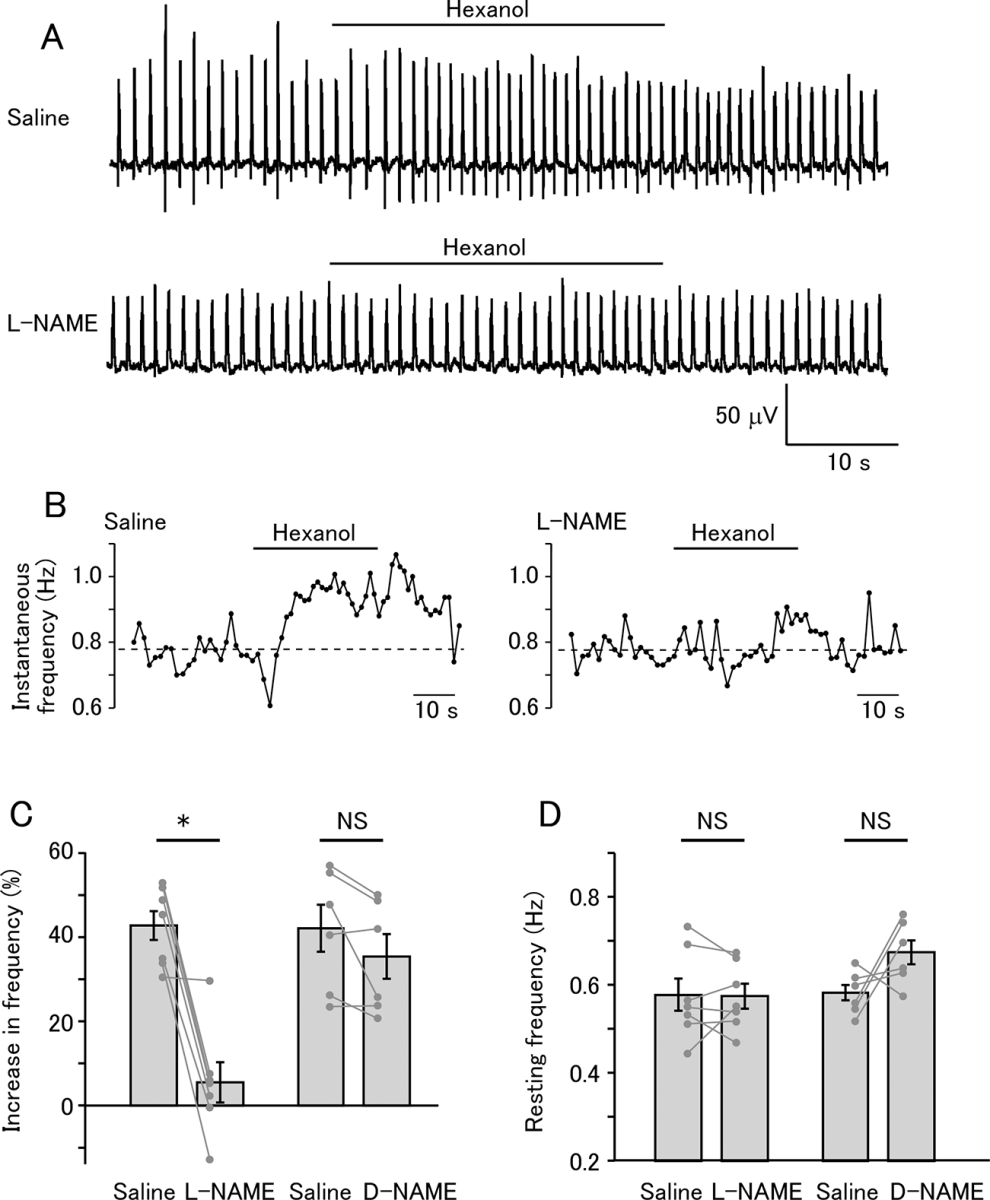
Odor-evoked NO release increases the frequency of the LFP oscillation in the PC lobe. (A) Ongoing LFP oscillation was recorded. During stimulation with hexanol, the LFP frequency increased (top). After application of L-NAME, the ongoing LFP oscillation was unaffected, but olfactory stimulation did not increase the LFP frequency (bottom). (B) Time course of the instantaneous frequency of the LFP oscillation in saline (left) and L-NAME (right). The dotted lines indicate the average of the pre-stimulus frequency. (C) Summary of the frequency changes by olfactory stimulation. Average and individual data are shown. L-NAME significantly reduced the frequency increase (*P<0.05; N = 7), whereas D-NAME did not significantly change the response (NS, not significant; N = 6). (D) Summary of the frequency of the resting LFP oscillation. Average and individual data points are shown. Neither L-NAME (N = 7) nor D-NAME (N = 6) significantly changed the frequency.

The regular oscillatory LFP was recorded from the PC lobe as previously reported [34]. Olfactory stimulation increased the frequency of LFP oscillation (Fig 2A, top and Fig 2B, left). After perfusion with L-NAME, the ongoing LFP oscillation was still intact, but the frequency increase in response to odor stimulation was significantly reduced (Fig 2A, bottom, and Fig 2B, right). Two-way mixed model ANOVA revealed a significant between-group difference between the L-NAME and D-NAME groups (P = 0.023). Post-hoc tests revealed that L-NAME significantly reduced the increase in the frequency (Fig 2C, left; saline 42.5±3.5%, L-NAME 5.4±4.8%, paired t-test with Bonferroni correction, P = 0.038, N = 7), and that D-NAME did not affect the frequency increase (Fig 2C, right; saline 41.8±5.9%, D-NAME, 35.2±5.9%, P = 0.226, N = 6). For the L-NAME group, Cohen’s d value was 3.36. The resting LFP frequency recorded before olfactory stimulation was not affected by either L-NAME or D-NAME (Fig 2D; between-group effect by two-way mixed model ANOVA, P = 0.185; for L-NAME group, saline 0.576±0.039 Hz, L-NAME 0.575±0.029 Hz, paired t-test with Bonferroni correction, P = 1.0, N = 7; for D-NAME group, saline 0.581±0.020 Hz, D-NAME 0.673±0.029 Hz, P = 0.224, N = 6). These results suggest that the odor-evoked frequency increase in the LFP oscillation is mediated by NO.

**Fig 3:**
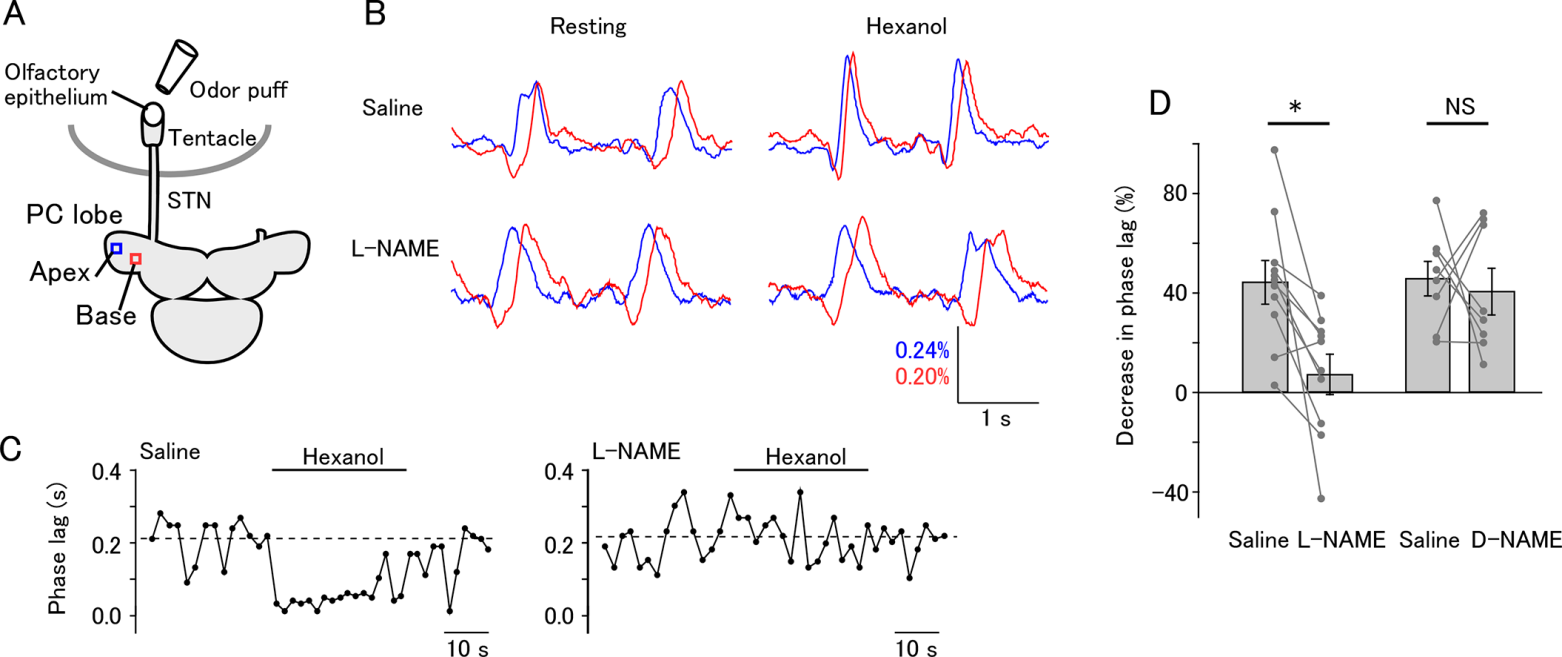
Odor-evoked NO release enhances spatial synchronicity of activity in the PC lobe. (A) Schematic of the experiment. Voltage imaging was made from the PC lobe in a tentacle-brain preparation stained with Di-4-ANEPPS. Apical and basal ROIs are shown. (B) Normalized fluorescence signals from the apical and basal ROIs. Before stimulation, the apical and basal signals had a lag (top left). The lag decreased during odor stimulation (top right). In the saline containing L-NAME, olfactory stimulation did not decrease the lag (bottom). (B) Time course of the phase lag in saline (left) and L-NAME (right). The dotted lines indicate the average of the pre-stimulus phase lag. (D) Summary of the responses of phase lag to odor stimulation. Average and individual data are shown. L-NAME significantly reduced the decrease in the phase lag (*P<0.05; N = 10), whereas D-NAME did not significantly change the response (NS, not significant; N = 8).

**Fig 4:**
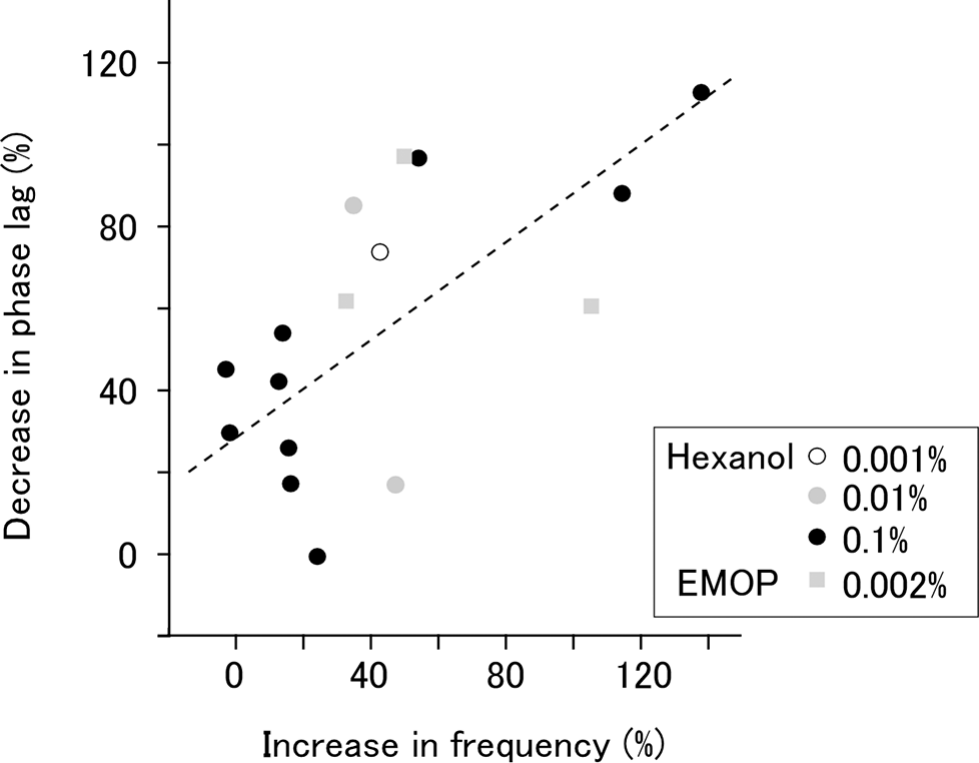
Correlation between changes in frequency and synchronicity. The decrease in phase lag was plotted against the increase in LFP frequency. Stimulation was made with hexanol at three different concentrations and EMOP at one concentration. The dotted line indicates least square fitting of all the data points. Spearman's rank correlation coefficient was 0.638 (P<0.01; N = 16).

The ongoing oscillatory activity of the PC lobe has a phase gradient along the apex-base axis. We performed voltage imaging of the PC lobe and analyzed the optical signal at two ROIs, one at the apex and one at the base, and examined whether the odor-evoked change in the lag is mediated by NO (Fig 3A). In normal saline, the phase lag between the apical and basal sites decreased in response to olfactory stimulation (Fig 3B, top and Fig 3C, left). The odor-induced decrease in the phase lag was reduced in L-NAME (Fig 3B, bottom and Fig 3C, right). Two-way mixed model ANOVA revealed a significant between-group difference between the L-NAME and D-NAME groups (P = 0.039). Post-hoc tests revealed that L-NAME significantly reduced the increase in the frequency (Fig 3D, left; saline 44.5±8.5%, L-NAME 7.4±7.9%, paired t-test with Bonferroni correction, P = 0.014, N = 10), and that D-NAME did not affect the odor-evoked decrease in the lag (Fig 3D, right; saline 46.1±6.5%, D-NAME 41.0±8.8%, P = 1.0, N = 8). For the L-NAME group, Cohen’s d value was 1.48. These results indicate that odor-induced reduction in the phase lag is also mediated by NO. Analysis of pooled data from experiments using various concentrations of hexanol and EMOP revealed that the degree of change in the frequency and synchronicity were correlated (Fig 4; Spearman’s correlation coefficient 0.638, P = 0.0078, N = 16).

**Fig 5:**
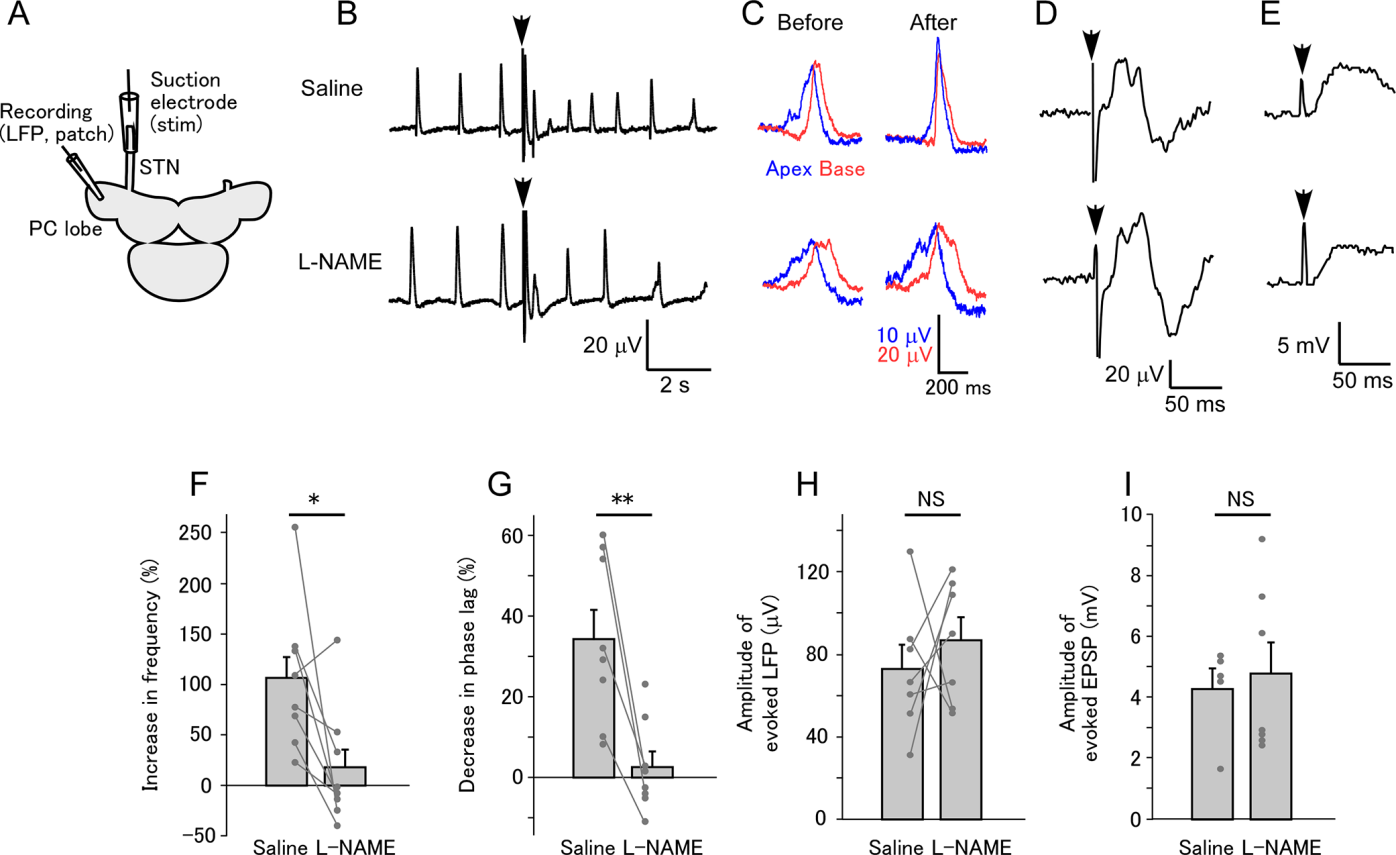
Electrical stimulation of the STN evokes NO-dependent responses. (A) Schematic of the experiment. A single electrical pulse was applied to the STN from a suction electrode. (B) STN stimulation (arrow) transiently increased the frequency of LFP oscillation (top). L-NAME blocked the frequency increase (bottom). (C) Modulation of the phase lag between the apical and basal recording sites. In normal saline, the lag decreased after STN stimulation (top). In saline containing L-NAME, STN stimulation did not change the lag (bottom). (D) The amplitude of the evoked LFP immediately following the stimulation in normal saline (top) did not change after incubation with L-NAME (bottom), suggesting that fast synaptic transmission to the PC lobe is intact in the presence of L-NAME. (E) The evoked EPSP was recorded in NB neurons in normal saline (top) and L-NAME (bottom). The amplitudes of the evoked EPSP were similar under these two conditions. (F) Summary of the changes in the frequency of LFP oscillation. Average and individual data points are shown in this and subsequent graphs. The LFP oscillation increased in response to STN stimulation, and this was blocked by L-NAME (*P<0.05; N = 10). (G) Summary of the changes in the phase lag between the apical and basal recording sites. The data connected by the lines are from the same samples. The phase lag decreased following STN stimulation, and this was blocked by L-NAME (**P<0.01; N = 8). (H) Summary of the amplitude of the evoked LFP. The amplitude did not significantly change after incubation with L-NAME (NS, not significant; N = 7). (I) Summary of the amplitude of the evoked EPSP in NB neurons. The amplitude did not significantly differ between saline and L-NAME groups (N = 5 for control and N = 7 for L-NAME).

Olfactory signals are transmitted to the brain via the STN. This suggests that stimulation of the STN also evokes responses similar to those by olfactory stimulation, possibly in a more reproducible way. In order to clarify this point, we stimulated the STN with a single electrical pulse (Fig 5A). STN stimulation increased the LFP frequency (Fig 5B, top). Bath application of L-NAME reduced the stimulus-induced increase in LFP frequency (Fig 5B, bottom and Fig 5F; saline 106.1±25.8%, L-NAME 17.3±20.1%; paired t-test, P = 0.033, N = 8). STN stimulation decreased the phase lag between the apical and basal recording sites (Fig 5C, top). L-NAME reduced the stimulus-induced decrease in the phase lag (Fig 5C bottom and Fig 5G; saline 34.2±7.3%, L-NAME 2.5±4.0%, unpaired t-test, P = 0.0029, N = 8 for saline and N = 8 for L-NAME). These results suggest that a single STN stimulation triggers a release of NO in the PC lobe that is enough to modulate the network activity.

The results above suggest that NO released by olfactory stimulation modulates the oscillatory activity of the PC lobe, but there remains the possibility that L-NAME blocks transmission of olfactory signals to the brain by reducing the resting NO level, rather than blocking the stimulus-evoked NO release in the PC lobe. Therefore, we tested whether L-NAME affects synaptic transmission from the STN to the PC lobe. STN stimulation evoked an LFP with a short latency following the stimulation (Fig 5D, top). L-NAME did not have any effect on the amplitude of the evoked LFP (Fig 5D, bottom and Fig 5H; saline 72.9±11.9 μV, L-NAME 86.9±11.2 μV, N = 7; paired t-test, P = 0.528). Since the evoked LFP may involve both presynaptic and postsynaptic elements, we made perforated patch recordings in NB neurons. STN stimulation evoked an EPSP in NB neurons, as shown previously [46]. The amplitude of the EPSP was similar in normal saline and L-NAME (Fig 5E and Fig 5I; saline 4.26±0.67 mV, N = 5; L-NAME 4.77±1.03 mV, N = 7; unpaired t-test, P = 0.686), further confirming that the synaptic transmission from the STN to the PC lobe is not affected by L-NAME.

## Discussion

In the present work, we obtained direct evidence for odor-induced NO release in the PC lobe. Gelperin [29] showed that L-NAME completely blocks LFP oscillation at 20 mM. We used a lower concentration of L-NAME, which we found to have little effect on ongoing oscillatory activity but almost completely blocked odor-induced modulation. These results suggest that NO is involved in both ongoing oscillation and odor-induced modulation, but these two effects have different sensitivity to NO.

Previous behavioral experiments [31,32] used the same amount of L-NAME per body weight as used for the bath solution in the present work. If we assume that the L-NAME concentration in the hemolymph is the same as in the present work, the resting LFP oscillation should be normal but its modulation by olfactory input should be blocked in those behavioral experiments. This assumption has been validated by the observation that the LFP oscillation was normal immediately after isolation of the brain from L-NAME-injected animals, but modulation of LFP oscillation by STN stimulation was blocked (Fig 3 in [31]). The modulatory effects of NO will thus be considered to be the key mechanisms of odor discrimination [32] and appetitive olfactory learning [31].

We clarified that NO modulates two aspects of network oscillations, frequency and synchronicity. Although the mechanisms to synchronize LFP oscillation are not well understood, the correlation between the changes in these parameters may suggest a simple common dynamical mechanism downstream of NO. Transient enhancement of synchronicity has also been shown by NO uncaging [6]. However, further experimental analysis will be required to identify the mechanisms of dynamical change during olfactory stimulation that simultaneously enhance frequency and synchronicity.

The NB neurons of the PC lobe presumably encode odor identity. Increased LFP frequency is accompanied by higher frequency of inhibitory synaptic input to NB neurons from B neurons, and decreases the firing rate of NB neurons [47]. During olfactory processing, such effects may serve to enhance the contrast of sensory representation by suppressing weak background firing. This may explain the impairment of odor discrimination in L-NAME injected slugs [32]. The functions of odor-evoked synchronization of the activity are more difficult to speculate about, because of absence of detailed single cell analysis. It may, however, change the interaction of neurons in the PC lobe. Spike-timing dependent plasticity takes place in a time window of about 50 ms [48], and NO presumably decreases the time lag between separate regions of the PC lobe to fit into this window. Yabumoto et al. [31] reported impaired olfactory learning in L-NAME injected slugs, suggesting involvement of NO in synaptic plasticity, although NO may also have biochemical, rather than dynamical, functions. Another possibility is that synchronicity affects summation of the synaptic potential on neurons that receive input from PC neurons. When the phase lag decreases, NB neurons will be more likely to fire synchronously, and this may result in a larger depolarization in the output neurons, which might lead to behavioral responses.

**Fig 6:**
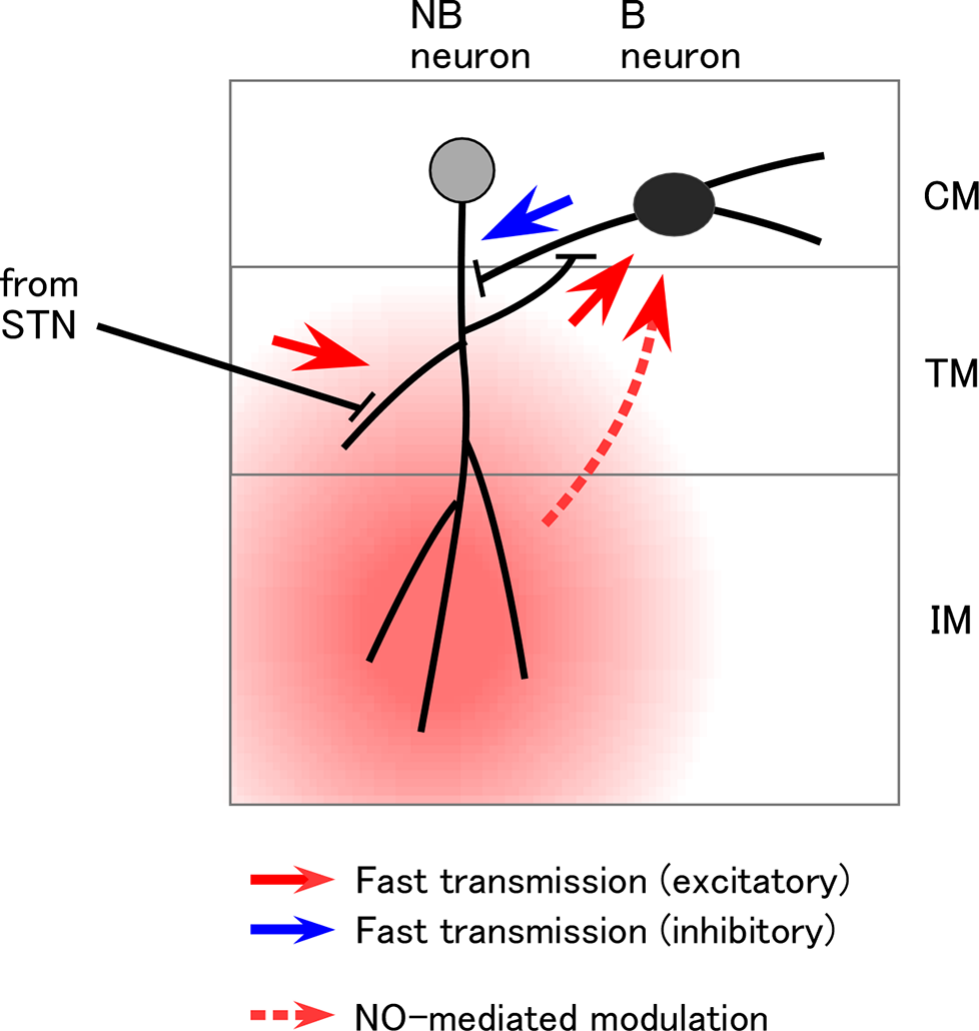
Schematic of the pathways that transmit olfactory information to the PC lobe. The PC lobe neurons (B and NB neurons) have somata in the cell mass (CM). Afferent fibers project in the terminal mass (TM) to make synapses on the NB neurons. NB neurons produce spikes that propagate afferently to activate synapses on B neurons. At the same time, spikes also propagate efferently into the internal mass (IM), where NO will be released. NO diffuses into the cell mass to depolarize B neurons, which modifies the network activity, presumably with the main effect of suppressing NB neurons.

Although the response of the NO concentration recorded by the NO electrode was slow, STN stimulation evoked rapid NO-dependent effects, suggesting potential roles of NO in local signaling at the sub-second time scale. We found that even a single electrical stimulation of the STN increases the LFP oscillation frequency and enhances synchronicity. These NO-dependent effects followed the early evoked LFP and EPSP in NB neurons, which were insensitive to L-NAME. This suggests that L-NAME blocks NO release from either NB neurons or downstream components. Given the morphology of PC lobe neurons [49], the expression pattern of NOS gene and the distribution of NADPH diaphorase activity, NB neurons seem to release NO in response to olfactory stimulation (Fig 6). Rather curiously, NADPH diaphorase activity is highest in the internal mass of the PC lobe [6,27,28]. The internal mass is situated about 50−100 μm away from the cell mass in which B neurons (which are the target of NO) extend neurites. Segregation of the NO source and target may improve the fidelity of signal transmission [50]. However, this will cause a slight time delay in the action of NO, which we estimate to be 0.1−0.5 s (time to half maximum concentration, using the diffusion coefficient of NO of 2.6×10^3^ μm^2^/s [51]). Delayed activation of NO-mediated modulation may result in biphasic response characteristics, with a fast inaccurate odor representation and a slow accurate representation. This could enable adaptive olfactory processing involving a tradeoff between accuracy and speed [52].
